# Drug-resistant tuberculosis in children: from global estimates to urgent action

**DOI:** 10.1183/13993003.01309-2026

**Published:** 2026-10-22

**Authors:** H. Simon Schaaf, Giovanni Sotgiu, Sabine E. Verkuijl

**Affiliations:** 1 Desmond Tutu TB Centre, Department of Paediatrics and Child Health, Faculty of Medicine and Health Sciences, Stellenbosch University, Cape Town, South Africa; 2 University of Sassari, Sassari, Italy; 3 Department for HIV, Tuberculosis, Hepatitis and Sexually Transmitted Infections, World Health Organization, Geneva, Switzerland

## Abstract

Awareness of childhood tuberculosis (TB) has increased in the past two decades. Children and young adolescents (those aged <15 years) bore 11% of the estimated global incident TB burden of 10.7 million cases in 2024, and a disproportionate 14.2% of TB deaths [1], with fatal outcomes occurring mainly in undiagnosed and inappropriately treated children [2].

## Shareable abstract

**New estimates suggest a substantial burden of drug-resistant tuberculosis in children and young adolescents. This editorial proposes an action plan to address gaps in prevention, case detection and treatment of drug-resistant TB in these age groups.** https://bit.ly/4bxoP0M

Awareness of childhood tuberculosis (TB) has increased in the past two decades. Children and young adolescents (those aged <15 years) bore 11% of the estimated global incident TB burden of 10.7 million cases in 2024, and a disproportionate 14.2% of TB deaths [1], with fatal outcomes occurring mainly in undiagnosed and inappropriately treated children [2]. Previous estimates for childhood multidrug-resistant (MDR)/rifampicin-resistant (RR)-TB have varied between 25 000 cases in 2014 [3], with modelling based on a systematic review, and 32 000 cases in 2016 [4], using a mathematical model of drug-resistant (DR)-TB patterns from global DR-TB surveillance projects. Based on 2021 Global Burden of Disease data from 204 countries, estimates have been updated for incident MDR/RR-TB (excluding extensively drug-resistant (XDR)-TB, defined as MDR/RR-TB plus resistance to fluoroquinolones and second-line injectable agents, according to the definition prior to 2021) and XDR-TB cases to 32 515 (95% uncertainty interval (UI) 20 968–51 288) and 1193 (95% UI 806–1814), respectively, accounting for 4.3% and 0.2% of the estimated approximately 759 300 paediatric TB cases in 2021, respectively [5].

Although children may be clinically less important in spreading *Mycobacterium tuberculosis* in the community, the epidemiological indicator of childhood incidence rate reflects TB dynamics, representing recent transmission, especially for children aged <5 years. However, young adolescents (those aged 10–14 years) with adult-type pulmonary TB might be a source of DR-TB themselves, acquired following poor adherence to complex treatment regimens or being prescribed inappropriate treatment regimens. Furthermore, young children diagnosed with DR-TB may be the index patient (the person first identified with TB), leading to the diagnosis of an unidentified DR-TB source through reverse contact tracing [6]. Paediatric DR-TB should not be viewed as a niche condition, but rather as a sentinel event reflecting ongoing community transmission and weaknesses in adult TB control programmes.

Moreover, children with DR-TB represent one of the clearest indicators of persistent global inequities in access to timely diagnosis, effective treatment and preventive care, reflecting a failure not only of clinical recognition, but also of healthcare and surveillance systems, and political commitment.

Microbiological diagnosis of the disease and of the mycobacterial drug resistance pattern is challenging in this population group because of the difficulty in obtaining adequate samples and of the frequent paucibacillary nature of TB. Samples are often not routinely collected, especially at primary healthcare level, with the majority being referred to higher levels of care for further evaluation, which is often not affordable to caregivers. Under-detection (poor suspicion of the disease) and underdiagnosis of TB, and especially DR-TB, are therefore common. Children diagnosed during contact investigations or exposed to known DR-TB sources are now more frequently started on empirical DR-TB treatment (based on the drug resistance pattern of the index case), although many clinicians still inappropriately wait for microbiologic confirmation. However, children with a presumed DR-TB diagnosis based on contact with a person with DR-TB may not necessarily be notified as DR-TB cases, leading to underreporting of DR-TB in this age group. In 2021 globally, out of 139 138 people with MDR/RR-TB who were started on second-line treatment, only 5506 (16.3% of the estimated 33 708 children with MDR/RR- or XDR-TB) were aged below 15 years. This figure has hovered between 3200 and 5600 from 2018 to 2024, suggesting there has been no progress in treatment coverage. The latest figure for 2024 was 4182 (3%) of all patients started on second-line treatment [1].

In the study reported by Liu *et al.* [5] in this issue of the *European Respiratory Journal*, incident cases, deaths, and age-standardised incidence and mortality rates in children aged <15 years with MDR/RR-TB and XDR-TB were analysed using the Global Burden of Disease 2021 database. Data were compared by age, sex, region, country and HIV/AIDS status. This study shows an increasing proportion of childhood MDR/RR-TB in the total TB burden, suggesting the limited impact of paediatric TB treatment and the systemic disruptions in TB prevention and care services during the COVID-19 pandemic, which disproportionately affected children [7, 8]. Dedicated action and further data collection and modelling are needed to address this trend. In table 1 we have included some suggested solutions along the cascade of care.

**TABLE 1:** Suggested actions to address drug-resistant tuberculosis (TB) in children

Suggested action	Comments
**TB vaccination**	Bacille Calmette-Guérin prevents severe forms of TB and some TB cases, but more effective vaccines are needed
**Preventing acquired and transmitted DR-TB in adults**	Early diagnosis of TB, both DS and DR-TB in adults, starting effective and shorter regimens, and supporting adherence to treatment cure or completion
**DR-TB TPT in contacts of DR-TB source patients**	Levofloxacin as TPT has been shown effective in MDR/RR-TB contacts and is recommended by WHO Ongoing study to evaluate delamanid as TPT in contacts of MDR/RR-TB as well as pre/XDR-TB contacts [20]
**HIV prevention and treatment**	Children with HIV are at higher risk of DR-TB Prevention of perinatal transmission of HIV and/or early effective antiretroviral treatment children diagnosed with HIV
**Contact investigation**	Contact investigation is important for identifying children eligible for TPT and those with presumptive TB Child contacts with TB disease of DR-TB source patients should be empirically treated according to the DST results of their identified source patient
**Reverse contact tracing**	Reverse contact tracing may lead to identification of microbiologically confirmed TB in an adult source patient while the child's microbiology may be negative
**Increased microbiological testing of TB in children**	Obtaining samples for microbiological testing in children at every level of care, using appropriate samples (including stool) and overcoming reluctance to obtain samples in young children
**Improved microbiological tests**	Microbiological tests with improved sensitivity, not only to confirm *M. tuberculosis* but also to do DST on obtained samples – at point of care as well as in laboratories
**Clinical diagnosis**	Improving clinical diagnosis of TB and increasing capacity and confidence of healthcare workers, including use of treatment decision approaches, with choice of regimen informed by the DST of the most likely source patient
**Integrating TB services into general healthcare**	Integration of TB services at the health facility and primary care level, *e.g.* nutrition, HIV, maternal and child health clinics may improve patient access, early diagnosis, and treatment outcomes
**Shorter and safer MDR/RR-TB treatment regimens**	Making use of shorter treatment regimens (6–9 months) as recommended by WHO Replacing more toxic drugs, *e.g.* linezolid, with newer, less toxic, oxazolidinone alternatives, which will need more rapid dosing and safety studies in children (see https://www.cheetafortb.org/) [21]
**Availability of MDR/RR-TB drugs for children**	Getting national TB programmes and manufacturers to produce and register child-friendly drugs for wider availability, but also to use adult formulations appropriately where child-friendly options not available [22]
**Recording and reporting of DR-TB cases**	Ensure notification of confirmed and clinically diagnosed DR-TB cases in children for accurate surveillance and planning
**Treatment outcomes**	Monitoring treatment outcomes and post-TB disease

DR: drug resistant; DS: drug susceptible; TPT: TB preventive treatment; MDR/RR: multidrug resistant/rifampicin resistant; pre/XDR: pre/extensively drug resistant; DST: TB drug susceptibility testing; WHO: World Health Organization.

It is important to highlight that, in the current study, children aged <5 years show a higher estimated incidence of drug-susceptible-, MDR/RR- and XDR-TB than those aged 5–9 and 10–14 years [5]. Young children are more commonly undiagnosed or underreported, leading to higher treatment coverage gaps in this age group [9]. TB should always be considered in the differential diagnosis, even with acute onset of symptoms in infants, young children and children living with HIV, who have a higher risk of progressing to disease.

Despite major advances in diagnostics and therapeutics, children remain systematically underrepresented in TB research, drug development and implementation programmes.

As expected, there was great variation in epidemiology in different geographical areas. A population-based study showed geographic variability in the burden of paediatric TB in the Western Cape of South Africa, emphasising the need to address local drivers to inform targeted interventions, which is also true for this global DR-TB study [10]. Gaps in the paediatric TB care cascade, from exposure to a person with *M. tuberculosis* to treatment completion, remain major concerns. Integrated data systems beyond TB treatment registers could improve estimates on TB epidemiology, allowing health system planning and, finally, improving outcomes for affected children and adults.

Addressing DR-TB in children relies on early and correct diagnosis of DR-TB and rapid onset of appropriate treatment, as most deaths are in children who are undetected, undiagnosed, not treated, or treated with inappropriate regimens [2]. Children with DR-TB can now also benefit from shorter (6- to 9-month) and more effective treatment regimens [11], addressing the issues of adherence, effectiveness, safety and tolerability.

DR-TB remains primarily a microbiological diagnosis, although its diagnosis in children often relies on the drug susceptibility test (DST) results of the infectious source. Age may play a role, with younger age children likely having higher concordance with DST patterns of their sources. Recent treatment decision algorithms for symptomatic children with presumed TB may be applied to DR-TB, with initiation of treatment based on the DST of the most likely source patient in children with risk factors for DR-TB who have a decision to treat for TB [12, 13]. Non-sputum-based tests for *M. tuberculosis*, such as urine lipoarabinomannan, and also near-point-of-care nucleic acid amplification tests (NAATs) on sputum or tongue swabs, do not provide DST results and the latter is not yet approved in children [14]. Low complexity automated NAAT (LC-aNAAT) on oral or tongue swabs have an extremely low yield in young children and should not be the primary confirmatory test [15]. LC-aNAAT (*e.g.* Xpert MTB/RIF Ultra) on stool samples does provide DST results for rifampicin and is an important child-friendly technique for the diagnosis of DR-TB (as part of the concurrent testing strategy recommended by the World Health Organization (WHO)). With the recent developments in targeted next-generation sequencing, these tests are becoming more available and feasible, although phenotypic testing is still necessary to confirm DST for some new drugs.

Recent disruptions in global funding for TB and HIV operational work, research and development will likely have a negative impact on aspects such as contact investigation, and TB and HIV prevention, as well as treatment, especially the supply of child-friendly formulations to high-burden TB countries [16]. National governments need to ensure an uninterrupted supply of drugs and continuing contact investigation and provision of TB and HIV prevention. Once-daily levofloxacin for 6 months is now strongly recommended by WHO since 2024, based on evidence from two randomised placebo-controlled MDR-TB prevention trials in children (TB-CHAMP) and adults (VQUIN) [17].

The uncertainty of clinicians in diagnosing TB and DR-TB based on a possible exposure to a DR-TB source patient remains a barrier to DR-TB treatment. The aforementioned shorter and safer treatment regimens may overcome this reluctance to initiate second-line treatment based on clinical diagnosis without microbiological confirmation [11].

With the appropriate management of childhood DR-TB, which includes optimised DR-TB regimens and consistent availability of drugs with (or without) child-friendly formulations, clinical outcomes are generally good (72–80% successful treatment) [18, 19]. This could be improved to >90%, as is the current WHO target, if optimal regimens are used and adherence to treatment is supported. The recent increase in bedaquiline resistance in several countries, a drug used in most current shorter regimens, is, however, of great concern, and development of new drugs, as well as timely evaluation of dose and safety in children, remains critically important.

The updated estimates of DR-TB in children and young adolescents based on the Global Burden of Disease Study strengthen our resolve to decisive action to detect these children so they can benefit from shorter and better treatment.

Reports of a growing burden of paediatric DR-TB signal that global TB control strategies are still failing the most vulnerable populations. Without investment in prevention, diagnostics, child-friendly therapeutics and surveillance, DR-TB in children risks becoming one of the most neglected infectious disease emergencies of the coming decade.
